# Prefrontal, Frontal, and Temporal Theta EEG Asymmetries and Self-Reports of Emotional Regulation

**DOI:** 10.7759/cureus.68771

**Published:** 2024-09-06

**Authors:** Mylorde Cherenfant, Merin Chandanathil, Raymond E Robinson, Richard M Millis

**Affiliations:** 1 Department of Medicine, College of Graduate Studies, American University of Antigua, St. Johns, ATG; 2 Department of Physiology, American University of Antigua, St. Johns, ATG; 3 Department of Clinical Medicine, American University of Antigua, St. Johns, ATG

**Keywords:** prefrontal theta asymmetry, temporal theta asymmetry, frontal theta asymmetry, medical school students, emotion regulation, quantitative electroencephalogram

## Abstract

Previous studies have shown that right-sided frontal alpha asymmetry (fAA) is an electroencephalography (EEG) marker for negatively valenced emotions and a marker for negative self-perceptions of a person's psychosocial interactions. Alpha activity is affected by the changes in visual stimulation associated with eye-opening and eye-closing; theta activity is not so affected. Therefore, this analysis investigates the relationship between an individual's theta asymmetry and self-perceptions of their psychosocial interactions. We used quantitative electroencephalographic (qEEG) data from eight right-handed male medical students aged between 19 and 38 years, recorded under eyes-open (EO) and eyes-closed (EC) conditions. Significant correlations were found between self-reported measures of psychosocial interactions via the Interactive Self-Report Inventory (ISI). The main finding was that greater left-sided frontal temporal asymmetry (fTA) under both EO and EC conditions was associated with lower “regulated” ISI scores and lower “dependent” ISI scores. Greater left-sided temporal theta asymmetry (tTA), under EC conditions, was associated with higher “anxious” ISI scores. Greater left-sided prefrontal theta symmetry (pfTA), under EO conditions, was associated with lower “relaxed” ISI scores. These findings suggest that theta asymmetries in the frontal, prefrontal, and temporal cortices may be indicative of negative emotional states. The results of this study underscore the potential of pfTA, fTA, and tTA to be used as biomarkers for cognitive-emotional balance. The implications for mental health interventions, particularly personalized therapeutic approaches, are significant.

## Introduction

The modern practice of electroencephalography (EEG) was born in 1924 with the research of Hans Berger, demonstrating the ability to record brain waves from the scalp [1]. However, it was not until 50 years later, in 1977, when it was proposed that the voltage amplitude of theta brain waves may be an indicator of changes in a person’s psychological state [2]. It was not until 2005 when it was reported that a control group exhibited greater left-sided temporal theta wave (4-7 Hz) EEG asymmetry than a comparison group of highly anxious individuals, wherein the theta voltage amplitudes recorded from the left temporal electrodes were significantly greater than those recorded from the right temporal electrodes [3]. It is now well-established that theta waves are associated with cognitive processes and emotional regulation [4]. Unlike alpha waves (8-12 Hz), theta waves are shown to exhibit more stable patterns across different states (eyes open (EO) vs. eyes closed (EC)) [5], perhaps making them a reliable measure for studying asymmetry and its relationship to neuropsychological functions. The brain's electrical asymmetry is thought to reflect the functional specialization of the cerebral cortex [6]. Frontal alpha asymmetry (fAA) is a well-established neurophysiological marker derived from quantitative EEG (qEEG) measurements, reflecting the difference in alpha wave (8-12 Hz) activity between the right and left frontal regions [7]. Previous studies have shown that right-sided fAA is an electrophysiological marker for negatively valenced emotions [8]. Higher left frontal alpha EEG voltage is associated with lower left and higher right frontal cerebral cortical activation [9]. A study from our laboratory suggests that fAA may be a marker for negative self-perceptions of psychosocial interactions in medical students [9]. We found that right-sided fAA was associated with "depressed" scores on the Interactive Self-Inventory (ISI) survey, a measure of a person's self-perceptions of their psychosocial interactions. However, alpha brain wave activity is affected by the changes in visual stimulation associated with eye-opening and eye-closing [10]; theta brain wave activity is not so affected [11]. Therefore, this analysis investigates the relationship between theta asymmetry and self-perceptions of psychosocial interactions in the same cohort of medical students as previously reported. Theta waves (4-7 Hz) are linked to cognitive functions such as memory, attention, and emotional processing [12]. Frontal, prefrontal, and temporal synchronization between theta and beta waves is shown to be an important determinant for coordinating emotional and cognitive functions [13]. Changes at the prefrontal, frontal, and temporal qEEG recording electrodes are known to reflect activity in the brain's Papez neurocircuitry for emotional regulation within the medial temporal lobe [14], a primary source of theta waves [15]. Increased alpha voltage indicates decreased activation or quiescence of the underlying cerebral cortex [16]. Greater right-sided frontal alpha asymmetry suggests reduced metabolic activity and signaling in the right (nondominant) frontal lobe, accompanied by increased activity in the left (dominant) frontal lobe [17]. Conversely, in the awake state, higher left-sided frontal theta voltage may be indicative of heightened metabolic activity and signaling in the left frontal lobe due to theta-beta coupling, a mechanism observed during cognitively demanding tasks that require the integration of multiple chunks of information [18, 19]. In this process, theta brain waves work in synchrony with increased beta activity, which is associated with greater cortical activation and enhanced information processing [20]. Therefore, for the present analysis, we hypothesized that left-sided prefrontal theta asymmetry (pfTA), frontal theta asymmetry (fTA), and temporal theta asymmetry (tTA) would correlate with a person's self-perceptions of their psychosocial interactions under both EO and EC conditions.

## Materials and methods

Eight right-handed male medical students aged between 19 and 38 years were the subjects of the present theta asymmetry analysis, based on a previously reported study of alpha asymmetry [9]. For this qEEG pilot study, the exclusion of females was done to reduce variability linked to the known effects of the menstrual cycle on fluctuations of alpha and theta wave activity. The qEEG parameters involving these bandwidths are strongly correlated with different phases of the menstrual cycle, driven by hormonal changes in estrogen levels [21]. These fluctuations can therefore affect the voltages, dominant frequencies, coherences, and asymmetries in brain wave activity, potentially acting as confounding variables if not precisely measured. Additionally, hormonal modulations have been shown to influence resting-state neural oscillations measured by magnetoencephalography (MEG), which are sensitive to phase-dependent changes during the menstrual cycle [22], further complicating the interpretation of brain activity patterns in mixed-gender studies without controlled hormonal measurements. Therefore, this exclusion strategy helped ensure more consistent and interpretable data, particularly in this early-stage pilot study research where controlling for hormonal variability would have been challenging without the inclusion of hormone monitoring protocols. Exclusion criteria based on socioeconomic, ethnic, and geographical origin factors were not considered; such factors were beyond the scope of this pilot study. The participants were healthy and under conditions of overnight fasting, without the use of alcohol, medications, or exercise for the prior 12 hours. Overnight fasting was employed in the experimental design to minimize the confounding effects of ingesting and metabolizing foods on the theta EEG bandwidth [23, 24].

All recordings were made at the same time of day, 9:00-10:00 AM. The EEG data were collected under EO and EC conditions. One participant's EEG data for the EO condition were excluded due to excessive recording artifacts. We analyzed the theta wave activity at the prefrontal sites (pf1 on the left and pf2 on the right), at the frontal sites (F7 on the left and F8 on the right), and at the temporal sites (T3+T5 on the left and T4+T6 on the right). Theta asymmetry was computed as mean voltage recorded at the left-sided prefrontal (pf1), frontal (F7), and temporal (T3+T5) electrodes minus mean voltage recorded at the right-sided prefrontal (pf2), frontal (F8), and temporal (T4+T6) electrodes. The ISI (New Mind Technologies, Inc., Roswell, GA) survey was used to measure psychosocial self-perceptions across 16 dimensions of psychosocial interactions, as previously described [9]. The ISI survey was administered within eight hours following the EEG recording session. Data were analyzed using Pearson’s product-moment correlation coefficient with a significance level set at p≤0.05.

## Results

Participant demographics, ISI scores, and theta asymmetries

All participants were right-handed, male, first-semester medical students who had, within one month prior to the study, matriculated to a Caribbean medical school from either the United States or Canada. The relevant demographics, ISI scores, and theta asymmetries are summarized below. Significant correlations between the theta asymmetries and ISI scores were found only for the "relaxed, regulated, dependent, and anxious" ISI scores. Each participant's theta asymmetries and ISI scores are presented in Tables 1-2. Table 1 summarizes the prefrontal, frontal, and theta asymmetries measured in each study participant. Table 2 summarizes the scoring of the 16 ISI dimensions for each study participant.

**Table 1:** Theta asymmetries in each participant Prefrontal, frontal, and temporal theta asymmetries were measured by quantitative electroencephalography (qEEG) under both eyes open (EO) and eyes closed (EC) conditions in each study participant, represented by the letters A-H. pfTA=fp1-fp2 prefrontal theta asymmetry; fTA=F7-F8 frontal theta asymmetry; tTA=[(T3+T5) –(T4+T6)] temporal theta asymmetry; N/A=data not available because of excessive artifacts in the EEG recording.

	A	B	C	D	E	F	G	H
pfTA EO (uV)	-9.0	-3.3	-7.5	-5.7	+3.8	N/A	+3.4	-0.1
pfTA EC (uV)	+3.0	+4.6	-0.3	+5.2	+4.4	+5.1	+2.4	+1.8
fTA EO (uV)	+26.4	-2.8	+19.0	+19.5	+76.5	N/A	+14.9	+31.9
fTA EC (uV)	+9.2	-0.2	+38.7	+44.1	+62.4	+32.8	+16.6	+29.1
tTA EO (uV)	+8.7	+70.1	+19.8	+20.0	+42.4	N/A	+18.4	+13.2
tTA EC (uV)	-8.5	+30.0	+10.2	+77.8	+41.4	+8.3	+14.1	-39.2

**Table 2:** ISI scoring for each participant Interactive Self-Inventory (ISI) scoring of the 16 ISI dimensions for each study participant, represented by the letters A-H.

ISI Dimension	A	B	C	D	E	F	G	H
Relaxed	30	26	25	26	20	22.5	22.5	26
Inhibited	15	12.5	11	23	15	16	15	12.5
Regulated	22.5	25.5	20	23	17	20	24	22
Impulsive	17	10	17.5	20.5	16	20	14.5	16.5
Passive	20	13	16	12	18	21	14	19
Assertive	25.5	25	21.5	24	22	23.5	23	27.5
Flexible	22.5	30	26	26	22	23.5	23	25
Perfectionist	27.5	15	13.5	25	18	20	22	13.5
Cooperative	30	27.5	28	19	22	20	22	25
Competitive	22.5	22	17.5	25	20	23.5	23	14
Independent	16	21	27	24	18	22.5	28	23
Dependent	26.5	25	24.5	22.5	18	22.5	24	26
Interactive	27.5	30	30	13.5	17	27.5	22.5	25
Avoidant	12.5	6	6	24	12	6	8.5	17.5
Anxious	7	8	8	9	10	7	7	6
Depressed	9	8	8	11	12	11	9	7.5

Participant A was 26 years old and exhibited the largest right-sided EO pFTA (-9.0 uV), the second largest left-sided EO fTA (+26.4 uV), and relatively small, equal amounts of left-sided EO tTA (+8.7 uV) and right-sided EC tTA (-8.5 uV). His theta asymmetries were associated with the highest "relaxed" (30/30) and the highest "dependent" (26.5/30) ISI scores.

Participant B was 38 years old and exhibited the largest left-sided EO tTA (+70.1 uV), the smallest right-sided EO pFTA (-3.3 uV), the smallest right-sided EO fTA, and EC fTA (-2.8 uV, -2.0 uV). His theta asymmetries were associated with the highest "regulated" ISI score (25.5/30).

Participant C was 33 years old and exhibited a relatively large right-sided EO pFTA (-7.5 uV) and virtually zero EC pFTA (-0.3 uV). His theta symmetries were associated with "relaxed, regulated, dependent, and anxious" ISI scores in the mid-range of the study group. 

Participant D was 35 years old, exhibiting the largest left-sided EC pfTA (+5.2 uV) and largest left-sided EC tTA (+77.8 uV), associated with relatively high "relaxed" (26/30) and "regulated" (23/30) ISI scores.

Participant E was 27 years old, exhibiting all left-sided theta asymmetries that included the largest left-sided EO fTA (+76.5 uV) and EC fTA (+62.4 uV) with relatively large left-sided EO tTA and EC tTA (42.4 uV, 41.4 uV). His theta asymmetries were associated with the lowest "regulated," the lowest "dependent," and the highest "anxious" ISI scores.

Participant F was 25 years old, whose EO measurements were not analyzable because of excessive EEG recording artifacts. All his EC theta asymmetries were left-sided, which included a relatively large left-sided EC pFTA (+5.1 uV) and the smallest left-sided EC tTA (+8.3 uV). His "relaxed, regulated, dependent, and anxious" ISI scores were unremarkable, mostly in the low or mid-range of the study group.

Participant G was 27 years old. All his theta asymmetries were left-sided and unremarkable, mostly in the mid-range of the study group. His "relaxed, regulated, and dependent" ISI scores were unremarkable, with a relatively low "anxious" score (7/30).

Participant H was 19 years old. His right-sided EO pfTA was virtually zero (-0.1 uV), and he had the smallest left-sided EC pfTA (+1.8 uV), also close to zero. He had the lowest "anxious" ISI score (6/30) with relatively high "relaxed" (26/30) and "dependent" ISI scores (26/30). His "Regulated" ISI score was in the mid-range of the study group (22/30). 

**Figure 1:**
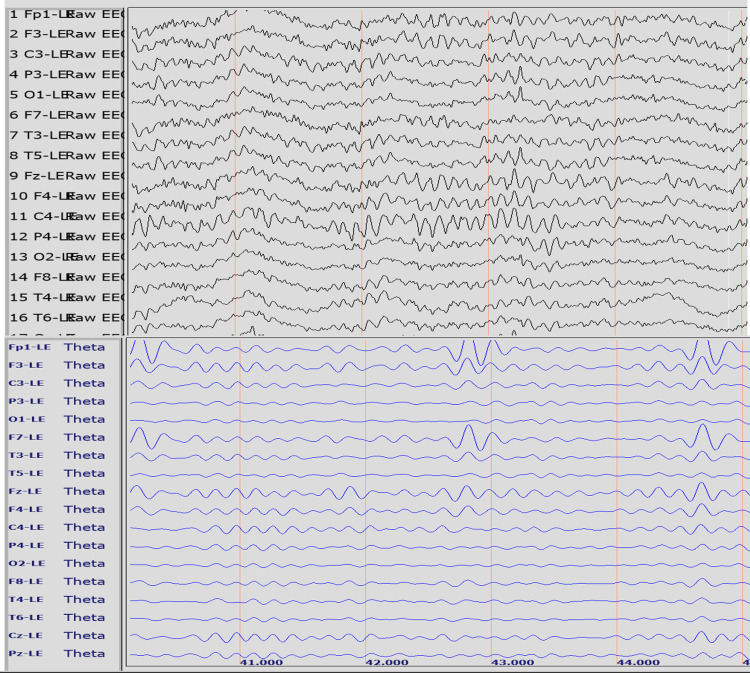
F7-F8 left-sided frontal theta asymmetry under the eyes-open condition Top panel: raw EEG; Bottom panel: raw EEG filtered to show only the theta (3-8 Hz) waves. Longitudinal red lines represent one-second marks for the same five-second recording period. Representative qEEG recording for subject E shows the largest amount of F7-F8 left-sided frontal theta asymmetry under the eyes-open (fTA +76 uV) condition. F7-F8 fTA is computed as the mean voltage difference measured at the standard F7 minus the standard F8 recording site. The markedly higher theta voltage amplitudes in the F7 than in the F8 recording channel result in a positive value for left-sided frontal theta asymmetry.

Figure 1 presents the raw (top panel) and filtered (bottom panel) EEG recordings from a representative participant (E). The recording demonstrates the difference in theta wave voltage between that recorded at F7 (larger voltage) and that recorded at F8 (smaller voltage) thereby producing a positive F7-F8 fTA. This participant exhibited the largest fTA during both the eyes-open and the eyes-closed conditions. 

**Figure 2:**
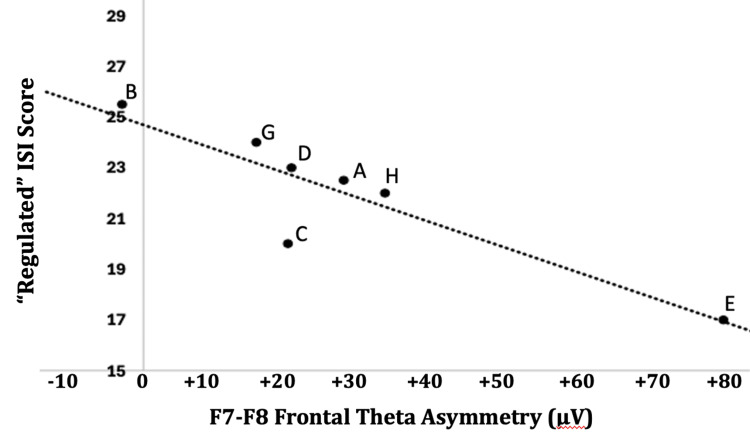
Relationship between "regulated" ISI score and frontal theta asymmetry measured with eyes open Subjects were eight right-handed males during a five-minute period of an eyes opened (EO) condition while visually focusing on a marked spot on an otherwise neutral wall in a dimly lit room, followed by completion of the Interactive Self-Report Inventory (ISI). The Pearson product-moment correlation coefficient was found to be r = -0.836, p = 0.009. The "regulated" ISI score was positively correlated with measures of approach and negatively correlated with measures of avoidance behaviors on the Beck Depression Inventory (BDI).

Significant correlations were found between left-sided fTA and negative self-perceptions under both the EO and EC conditions. Figure 2 shows the correlation based on findings for the EO condition (EO r= -0.836, p=0.009); the EC conditions result was similar with respect to the “regulated” ISI score (EC r= -0.896, p=0.006).

There was a significant negative association (r = -0.791, p = 0.019) between left-sided fTA and the “dependent” ISI score, suggesting that the participants exhibiting greater left-sided fTA, only under the EC condition, may have responded to the ISI survey with lower feelings of "dependent." A significant positive correlation between the ISI "anxious" score and left-sided tTA was found only under the EC condition (r = +0.832, p = 0.01). This finding suggests that higher left-sided tTA was associated with increased "anxious" perceptions of the participants' psychosocial interactions. A significant negative association between pfTA and the “relaxed” ISI score (r = -0.832, p = 0.02) indicates that the participants exhibiting greater left-sided pfTA under the EO condition responded to the ISI survey with lower levels of feeling "relaxed" with respect to the self-perceptions of their psychosocial interactions.

## Discussion

This study is the first to report that greater left-sided pfTA, fTA, or tTA may be associated with self-report survey scores indicative of emotional health in a cohort of individuals without a diagnosis of mental illness. The present analysis explored the relationship between prefrontal, frontal, and temporal theta brain wave asymmetry and self-perceptions of psychosocial interactions in a cohort of medical students. The study hypothesis was supported by the findings that the scores for four of the 16 ISI dimensions showed significant correlations between self-perceptions of the participants’ psychosocial interactions and their left-sided theta asymmetries. Greater left-sided pfTA, fTA, and tTA were associated with lower "regulated," lower "relaxed," and higher “anxious” ISI scores. These scores are reported to be significantly correlated with measures of approach-avoidance behaviors on the Beck Depression Inventory (BDI) and indicative of negative self-perceptions of a person’s psychosocial interactions [9]. The association between left-sided fTA and low “dependent” ISI score appears to be an anomaly because low dependence is more likely to be an indicator of positive, rather than negative, self-perceptions of one’s psychosocial interactions.

The ISI survey is designed to assess various aspects of emotional balance, which may be correlated with qEEG results, to tailor neurotherapy treatments. Based on the approach-avoidance paradigm, ISI scores, which are indicative of negative self-perceptions of a person’s psychosocial interactions, are shown to be highly correlated with “avoidance” responses on the BDI scale [9]. The "regulated" scores on this survey may be important in understanding an individual's emotional self-regulation capabilities. The low-regulated scores, which were, in the present analysis, found to be correlated with greater left-sided fTA under both the EO and EC conditions, suggest difficulties in emotional regulation. Low-regulated scores are often associated with impulsivity and poor self-control. The high "anxious" scores on the ISI survey, shown to be significantly associated with greater left-sided tTA only with EC, may indicate that an individual experiences frequent or intense anxiety. Low “relaxed” ISI scores, shown in the present study to be significantly associated with greater left-sided pfTA, only under the EO condition, suggest that an individual may struggle to feel calm and relaxed with eyes open. The "dependent" ISI scores, which appear to be anomalous with respect to a person’s self-perceptions of their psychosocial interactions, may provide insights into an individual's reliance on others for emotional support and decision-making. The low dependent scores, which were significantly associated with greater left-sided fTA only under the EC condition, suggest a high level of emotional independence. The same group of individuals with high left-sided fTA with both EO and EC gave ISI survey responses indicative of low emotional self-regulation. However, those with high left-sided fTA only with EC (not with EO) gave ISI responses indicative of low emotional dependence, an indicator of positive (not negative) self-perceptions of their psychosocial interactions. These findings suggest the novel hypothesis that fTA may be a rather fluid measurement that could be affected by visual inputs, consistent with what is known about the changes in frontal theta activity associated with learning and memory tasks.

Frontal and temporal asymmetry and self-regulation

The significant correlations identified in this analysis between negative self-perceptions and both frontal and temporal theta asymmetry provide compelling evidence for a neurobiological basis reflected in the qEEG signature of frontal and temporal theta asymmetry underlying interindividual differences in emotional control. These findings have potential implications for understanding the neural mechanisms that influence self-perceptions in various psychosocial contexts. The observed right-sided frontal theta symmetry correlating with negative self-perceptions, specifically under both EO and EC conditions, suggests a robust association between theta activity and emotional regulation. Theta waves, oscillating at 4-7 Hz, are known to be involved in cognitive functions such as memory, attention, and emotional processing. The significant negative correlations with the "regulated" and "dependent" self-perception scores under eyes-open and eyes-closed conditions imply that increased right-sided frontal theta asymmetry is linked to feelings of dysregulation and independence from external influences. This discordance supports the hypothesis that theta asymmetry could be an indicator of emotional dysregulation, which aligns with prior research on alpha asymmetry and negatively valenced emotions.

Temporal theta asymmetry and anxiety

The significant positive association between left-sided tTA and increased anxiety levels further underscores the role of theta activity in emotional processing. The temporal EEG electrodes are positioned near the medial temporal lobe and the Papez circuit, both critical areas for emotional control [25]. The findings that individuals exhibiting higher tTA under eyes-closed conditions correlate with higher "anxious" responses on the ISI suggest that temporal theta activity may be intricately linked with the neural circuits involved in anxiety regulation. This mirrors findings from studies on anxiety and brain activity, reinforcing the concept that asymmetrical theta activity can reflect emotional states and traits [3].

Prefrontal theta asymmetry and relaxation

The observed significant negative correlation between individuals exhibiting prefrontal theta symmetry under eyes-open conditions and the "relaxed" self-perception score indicates that lower relaxation levels may be associated with higher pfTA. This suggests that theta activity in the prefrontal cortex, an area involved in executive functions, emotional regulation, and response control [26], may play a role in how individuals perceive their emotional states. The prefrontal cortex's involvement in top-down control of emotions provides a neurobiological framework for understanding these findings, suggesting that increased left-sided theta asymmetry may impair the ability to maintain relaxed and calm states.

Implications for mental health interventions

The significance of theta asymmetry in persons affected by mental illness is highlighted by its relationship with treatment outcomes reported in patients diagnosed with clinical depression. Previous studies have shown that individuals with right-sided fTA respond better to antidepressant treatments, such as escitalopram [27]. The significance of this finding is based on the known connectivity between the anterior cingulate cortex and the dorsolateral prefrontal cortex involved in regulating emotional responses and cognitive processes [27]. Theta activity in these areas is thought to reflect the degree of engagement in top-down cognitive control, which is necessary for managing emotional conflicts and negative affective states often experienced in depression [28]. Less theta power on the right is equivalent to more theta power on the left, indicating left-sided fTA. This phenomenon is reported to be positively correlated with depressive symptoms [28]. The participants in the present study were eight healthy medical students without histories of mental illness or neuropsychiatric disease. The aforementioned findings about left-sided fTA [27, 28] appear to corroborate our finding that the participants' "regulated" ISI scores were significantly negatively correlated with left-sided fTA under both eyes-open and eyes-closed conditions. Additional corroboration is reflected in our finding that greater left-sided pfTA significantly negatively correlated with "relaxed" only with eyes opened and greater left-sided tTA significantly positively correlated with "anxious" ISI scores, albeit only with eyes closed. These findings suggest theta asymmetry as a potential indicator of susceptibility to depressive symptoms in individuals in the absence of a depression diagnosis. The identification of theta asymmetry as a potential marker for emotional dysregulation also opens new avenues for personalized therapeutic approaches. For example, neurofeedback training that targets imbalances in theta asymmetry could help individuals better regulate their emotions and improve their psychosocial interactions. Therefore, the present findings may be generalizable to different populations, including those with varying needs for mastering psychosocial interactions, such as individuals with anxiety disorders, and depression, or those undergoing high-stress training programs like medical students.

Limitations and future directions

While this study provides valuable insights, it is limited by the small sample size of eight right-handed male medical students. Future research should aim to replicate these findings in larger and more diverse populations to enhance the generalizability of the results. Additionally, longitudinal studies could help elucidate the causal relationships between theta asymmetry and self-perceptions of psychosocial interactions. Exploring interventions that can modulate theta asymmetry and examining their effects on emotional regulation and psychosocial interactions would be a fruitful area for future research.

## Conclusions

In summary, this analysis demonstrates that left-sided theta asymmetry, particularly in the frontal and temporal regions, may be significantly correlated with negative self-perceptions of psychosocial interactions among medical students. These findings provide evidence for a neurobiological basis for interindividual differences in emotional control and highlight the potential of theta asymmetry as a marker for emotional dysregulation. The implications for personalized mental health interventions and the potential for generalizing these findings to broader populations underscore the importance of further research in this area.
